# The Other Side of the Bedside

**DOI:** 10.1177/2374373519862929

**Published:** 2019-07-10

**Authors:** Jessica A Schmitt

**Affiliations:** 1 Department of Pediatrics, 9968 University of Alabama at Birmingham, Birmingham, AL, USA

**Keywords:** clinician–patient relationship, communication, challenges, empathy, relationships in health care, rounding, trust, team communication

“You look just like your sisters.” I have heard this countless times, and it always makes me smile. A teacher, doctor, and businesswoman. Despite similar childhoods, we found very different careers. My big sister Jenny taught me to read when I was 3 and takes credit for me becoming a doctor. As her first pupil, I take credit for turning her into a teacher. In summer 2016, Jenny had an ordeal that left lasting changes on us all and significantly impacted how I approach my job.

In June 2016, I was in the second year of pediatric residency, and Jenny was pregnant. As her due date passed, the Schmitt clan anxiously waited for news. Finally, at 8:30 on June 21, my brother-in-law called to say that following a prolonged induction and caesarian section, my niece Alexandra Kate had joined the world. Jenny didn’t come to the phone.

I was distracted on rounds and for the rest of the day, waiting for more updates. By that afternoon I felt something was wrong. We Schmitt sisters are close; although I knew Jenny would be tired, I knew this lack of contact was atypical for her. A call to the younger sister confirmed she knew no more than I. Eventually mom called, telling me Jenny had HELLP (hemolysis, elevated liver enzymes, low platelets) syndrome. I told mom not to look up anything online, knowing that Wikipedia cites a 1% mortality and 25% morbidity (1).

The following day was a blur. After my 28-hour shift, I finally came home. I slept for a few hours and woke, quickly reaching for my phone. Standing alone in my apartment, I listened to the voicemail and my heart broke. I heard the catch in my mother’s voice as she told me Jenny wasn’t improving, she had pressure syndrome, and they were anticipating dialysis. Who can fault my nonmedically trained mom for hearing “pressure” rather than PRES (posterior reversible encephalopathy syndrome)? Nomenclature was irrelevant; mom’s baby was sick, and she was scared.

I called my boss and 30 minutes after waking, I was in the car driving to get to Jenny. Seven hours and many cups of coffee later, I walked into a hospital room, not as a doctor, but as a sister for the first time in my life. What should have been a joyful moment meeting my niece was instead traumatic. Jenny’s swollen hands reached for mine and her yellow eyes showed none of their typical brightness. I held her hand and sat at her bedside, on the wrong side of the bedside.

I am a physician, I stand on the patient’s right side to better feel the liver. I stand on the side closest to the door where I exit after completing my duty. I stand. I don’t sit. As a Jenny’s sister, I sat on her left. I sat on the side facing the door just in case a provider with information passed. I sat because I was too fatigued to stand. I sat because the worry I felt was so heavy, I couldn’t remain upright. I sat, helpless.

Sitting with Jenny, I became accustomed to her waxing and waning delirium. Once she was making small talk with a technician from the neurology team. The technician started to check Jenny’s orientation and asked her, “What month is it?”

“February!”

The technician was caught off-guard, then asked again, “What month is it?”

“February!” Jenny replied again with confidence.

“When did you have your baby?”

Without hesitation Jenny stated calmly and clearly, “I had my baby, Alexandra Kate, on June 21, 2016.”

“OK…then, what month is it?”

“FEBRUARY!” she replied. Jenny looked at me and smiled. I was born in February.

At her bedside, we waited for updates from Jenny’s medical teams. Eventually a diagnosis was made: atypical hemolytic uremic syndrome. The attending came in with her trail of trainees, sat down, and explained slowly and calmly without medical jargon what happened and what the plan would be. My parents, Jenny’s in-laws, and I hung on every word. When she was done, she asked each of us what questions we had; there were none. Her bedside manner was exemplary and could have been filmed as an example to teach medical students empathetic communication. As soon as she left, however, my parents and Jenny’s in-laws all looked to me and asked, “What did she say?”

I realized that what I saw as an empathetic, jargon-free, thoughtful explanation with adequate opportunity to ask questions, was a blur to my family. Although the doctor had explained the medical situation clearly, she had avoided the questions we didn’t want to ask: Will Jenny die? Will she need a kidney transplant? Is this genetic? The unasked, difficult questions went unanswered because we were too scared to ask, and the physician did not preemptively answer.

Six months later, I walked into the neonatal intensive care unit (NICU) expecting the typical routine as a third-year resident. Entering the NICU, hearing the mechanical beeps, seeing the ill patients, I immediately felt an overwhelming sense of dread and fear. The Schmitt sisters had had enough of critical care units. Memories of feeling helpless and stuck at Jenny’s bedside flooded me. I took a few breaths and tried to distance myself emotionally.

I went to see my patients. One had been in the NICU for many weeks, and her mother was at the bedside. I introduced myself and we began to chat. After so long in the NICU, the cause of prematurity is not often discussed, so I asked. I held back tears as the new mother explained her baby was born after she herself developed HELLP. I reached for her hand and asked how she was doing. Had I not recently been on the other side of the bedside, I would never have connected in that way with this mother. After we talked, the new mother thanked me for showing interest not just in her child but in herself as well.

Jenny’s story thankfully ends well. She is healthy and happy. Her life has reached a new normal and so has mine. While we have returned to our routine, Jenny’s illness and my experience at her bedside have changed me as a physician. Remembering what it is like on the other side of the bedside, I do not order frequent vitals unless they are absolutely necessary; sleep is hard to find in the hospital. I call the lab to ask if there is blood remaining to test for a needed lab before asking for a new blood draw. Those blood sticks hurt the patient and are difficult for the families to watch. I sit. I offer hugs. I am not perfect, but I focus on acknowledging the unasked questions even if I am unable to answer them.

As a pediatric endocrinology fellow, I have told dozens of parents that their child has type 1 diabetes, a life-changing diagnosis. When talking with them, I remember how it feels on their side of the bedside. I remember the trauma we endured when Jenny was sick, the fear of the unasked and unanswered questions, and the need for information to be repeated frequently and clearly.

As a doctor, today is my normal workday and the hospital is my normal workplace; to my patients and their families it is the worst time and place of their life. By recalling and addressing the fear on the other side of the bedside, we providers can strive to fulfill Dr Peabody’s ideal: “for the secret of the care of the patient is in caring for the patient” (2). As a pediatrician, I believe in going a step further; the secret in caring for the patient is caring for the family.
